# Comparison of 3R4F cigarette smoke and IQOS heated tobacco product aerosol emissions

**DOI:** 10.1007/s11356-021-18032-x

**Published:** 2021-12-22

**Authors:** Teemu Kärkelä, Unto Tapper, Tuula Kajolinna

**Affiliations:** 1 Department of Nuclear Energy, VTT Technical Research Centre of Finland Ltd., Kivimiehentie 3, 02044 VTT Espoo, Finland; 2 Department of Mobility and Transport, VTT Technical Research Centre of Finland Ltd., Tietotie 4c, 02044 VTT Espoo, Finland

**Keywords:** Combustible cigarette, Heated tobacco, Particulate matter, Volatile matter, Low emissions

## Abstract

In this study, the smoke from a 3R4F research cigarette and the aerosol generated by the Heated Tobacco Product IQOS, also referred to as the Tobacco Heating System (THS) 2.2 in the literature, were compared. The objective was to characterize the gas and suspended particulate matter compositions in the mainstream smoke from a combusted 3R4F cigarette and in the aerosol generated by IQOS during use. The results indicated that the determined aerosol emissions from IQOS were notably lower than in the cigarette smoke under a Health Canada Intense puffing regimen. As an interesting detail in this study, the maximum nicotine concentrations within a puff were practically the same in both the 3R4F smoke and the IQOS aerosol, but the average concentration was lower for the IQOS aerosol. For both products, water constituted a significant proportion of the particulate matter, although it was substantially higher in the IQOS aerosol. Furthermore, combustion-related solid particles observed in the 3R4F smoke contained elements such as carbon, oxygen, potassium, calcium, and silicon. In contrast, IQOS aerosol particulate matter was composed of semi-volatile organic constituents with some minor traces of oxygen and silicon. The particulate matter found in the IQOS aerosol was volatile, which was especially noticeable when exposed to the electron beam of the scanning electron microscope (SEM) and Transmission Electron Microscope (TEM).

## Introduction

Combustion is a complex phenomenon resulting in the formation of many chemical compounds through several mechanisms (Drysdale 1985). In the case of complete combustion of organic materials (e.g. biomass), the emissions result in the formation of carbon dioxide and water only (Simmons 1995). Complete combustion is only possible when adequate stoichiometry is achieved between the combustible material and the oxidizer. However, this condition is rarely met in real-life combustion scenarios. More specifically, when combustibles and oxidizers are not present in the same molar proportions and temperatures are not homogeneously distributed, or an overall excess of oxidizers are unavailable, incomplete combustion takes place (Caillat and Vakkilainen 2013). In most of the incomplete combustion processes of organic materials, several gas species and solid particles (soot) are emitted, including carbon monoxide, polycyclic aromatic hydrocarbons (PAHs), volatiles, semi-volatile organic compounds, and non-volatile organic and inorganic particulate matter (so called ‘tar’) (Tomasi and Lupi 2017; Stauffer et al. 2007). The gas species, condensed organic compounds and solid particles emitted from the combustion process together constitute smoke. Smoke also contains various minerals that have been taken up by living plants, such as calcium, potassium and magnesium. These minerals do not burn and form ash, also referred to mineral ash (Vassilev et al. 2013). In contrast to the processes occurring when organic materials are combusted, when a biomass is heated below a specific temperature threshold, representing the ignition point of a specific material, combustion mechanisms are not activated. Only distillation and low temperature pyrolysis processes take place, the latter corresponding to the thermal degradation of biomass, which occurs irrespective of the presence of oxygen. This results in the production of charcoal (solid), bio-oil (liquid) and fuel gases (Adaganti et al. 2019). When the emitted gases cool down, vapours condense to form droplets that, together with the gases, constitute a secondary aerosol that is not smoke. For example, pine cones and sunflower shells do not combust below 200 °C, and only pyrolysis takes place (Haykiri-Acma 2003). In the case of tobacco, the ignition temperature for combustion is approximately 400 °C (Barontini et al. 2013; Wang et al. 2005). Below the ignition temperature of a given biomass, only volatile and semi-volatile substances release into the gas vapour phase, which cool down and form suspended droplets that constitute an aerosol.

When comparing the smoke generated from biomass combustion and the aerosol formed during heating of the biomass below the ignition temperature, a clear distinction in their chemical composition and physical characteristics is observed. In fact, in the case a material is heated, the formation and emission of solid soot, ash, organic and inorganic particles are avoided (Stauffer et al. 2007; Tomasi and Lupi 2017). Atmospheric PM1 (particulate matter with aerodynamic diameter less than 1 µm), mainly comprised of solid particles originating from combustion, has been associated with similar health effects in humans to those of PM2.5 (Chen et al. 2017a, b). Inhalation of these non-soluble particles has been associated with the development of respiratory conditions including asthma and inflammation, which can lead to gene damage and cancer (Chen et al. 2017a, b). In addition to solid particles, appreciable quantities of polycyclic aromatic hydrocarbons (PAHs) are also part of the particulate matter emitted from combustion sources, as these compounds are emitted at the high temperatures associated with biomass combustion (exceeding 700 °C) (Tsekos et al. 2020). Atmospheric PAHs are a major threat to public health due to their mutagenicity and carcinogenicity (IARC 1983, 1984; Lewtas 2007). The common carcinogenic effect of PAHs related to human cells is observed as DNA damage through the formation of adducts in several organs, including liver, kidney and lungs (Vineis and Husgafvel-Pursiainen 2005; Xue and Warshawsky 2005). As the formation of PAHs are strongly associated with the high temperatures generated during combustion, the yield of PAHs would be minimal when a biomass is heated below the ignition temperature. Therefore, it is better to avoid combustion in order to minimize the adverse health effects in humans associated with the inhalation of combustion related particulate matter.

Similar to the atmospheric aerosols derived from combustion processes, cigarette smoke also contains solid particles and PAHs as part of its complex particulate matter. In the case of cigarette smoke which is formed from tobacco combustion, in addition to the products generated and emitted from complete and incomplete combustion processes, cigarette smoke also contains heavily pyrolyzed compounds owing to the thermal degradation of products occurring at the high temperatures generated during smouldering combustion in oxygen-lean conditions (Wójtowicz et al. 2003). As a result, cigarette smoke is a complex mixture of vaporised, pyrolyzed, and combusted products, which include traces of the minerals originally present in the soils where tobacco plants were growing (Rodgman and Perfetti 2013), PAHs (Vu et al. 2015) and viscous liquid or other oily materials—better known as tar (Hoffmann et al. 1997; Valavanidis and Haralambous 2001). For several decades, cigarette smoke has been known to produce adverse effects in humans (US Surgeon General 1989). The harmful chemicals found in cigarette smoke have been linked to several diseases such as lung cancer, heart disease, and emphysema (US Department of Health and Human Services 2014). The harmful chemicals in cigarette smoke originate mainly from the thermal decomposition of tobacco´s organic compounds under an oxidative atmosphere at the high temperatures associated with tobacco combustion, reaching up to 900 °C (Torikaiu et al. 2005; Baker 2006).

In recent years, the tobacco industry has shifted their focus towards the development of novel non-combusted tobacco products to replace conventional cigarettes, which significantly reduce the level of harmful emissions. Electrically heated tobacco products represent the latest generation of tobacco containing products (O’Connell et al. 2015). As part of this evolution, a new type of non-combustion tobacco product, that heats tobacco instead of burning it (also known as “heat-not-burn” or heated tobacco product—HTP), has been developed and commercialised in numerous countries by Philip Morris International (Smith et al. 2016). As HTPs operate at temperatures significantly lower than in combusted cigarettes, combustion is avoided, which results in a significant reduction in the levels of harmful chemicals released (Schaller et al. 2016; Cozzani et al. 2020). In order to initiate tobacco combustion, the temperature needs to reach around 400 °C (Barontini et al. 2013). As these products are intended as cigarette alternatives, it is important to evaluate their emissions in comparison to cigarette smoke. In fact, HTPs have the potential to reduce exposure to harmful chemicals and solid particles in consumers in comparison to cigarette smoke.

In this investigation, the 3R4F research cigarette from the University of Kentucky (3R4F 2019) and the IQOS heated tobacco product (Smith et al. 2016) were selected as representatives for conventional cigarettes and heated tobacco products, respectively. In general, it is well established that cigarette smoke and IQOS aerosol have a different physico-chemical nature (Schaller et al. 2016), but little is known about the particulate matter state (liquid or solid). Cigarette smoke has been studied for a few decades, and the examinations have resulted in detailed information demonstrating that smoke contains solid organic particles, which are generated and emitted during combustion. In particular, these particles are generally composed of oxygenated carbon-based material, solid elemental carbon and minerals, semi-volatile droplets and mineral ashes (Pratte et al. 2017). So far, only a few studies have been conducted to examine the physico-chemical properties of the aerosol produced by IQOS, since it has only been marketed commercially since late 2014. Pratte et al. (2017) demonstrated that no solid particles, compared to the background air, were observed in the aerosol generated from the use of IQOS. The observations were made based on the thermo-denuded IQOS aerosol and scanning electron microscopy imaging compared with the blank. In this context, airborne compounds exiting the filter end of this heated tobacco product need to be investigated further regarding their physico-chemical properties. In the current study, the gaseous and particulate matter emissions from the use of 3R4F cigarettes were compared to that of the aerosol produced from the IQOS heated tobacco product.

## Experimental approach

Smoke from 3R4F research cigarettes (3R4F 2019) and the aerosol generated by the HTP IQOS, also referred to the Tobacco Heating System (THS) 2.2, when used with a regular tobacco stick variant D2 (Smith et al. 2016) were studied. The 3R4F cigarette was chosen as a reference for conventional cigarettes (manufactured by University of Kentucky, Lexington, KY, USA). During cigarette combustion, temperatures are reaching up to 900 °C (Baker 1975), releasing smoke that contains nicotine and thousands of other chemicals (Rodgman and Perfetti 2013). IQOS is a heated tobacco product (manufactured by Philip Morris International) operating at a maximum heating blade temperature of ca. 350 °C, thus resulting in a maximum tobacco stick temperature of ca. 320 (Cozzani et al. 2020). In this condition, nicotine and flavours are released while the tobacco temperature is kept sufficiently low to avoid combustion (Smith et al. 2016; Cozzani et al. 2020).

### Experimental setup

**Fig. 1:**
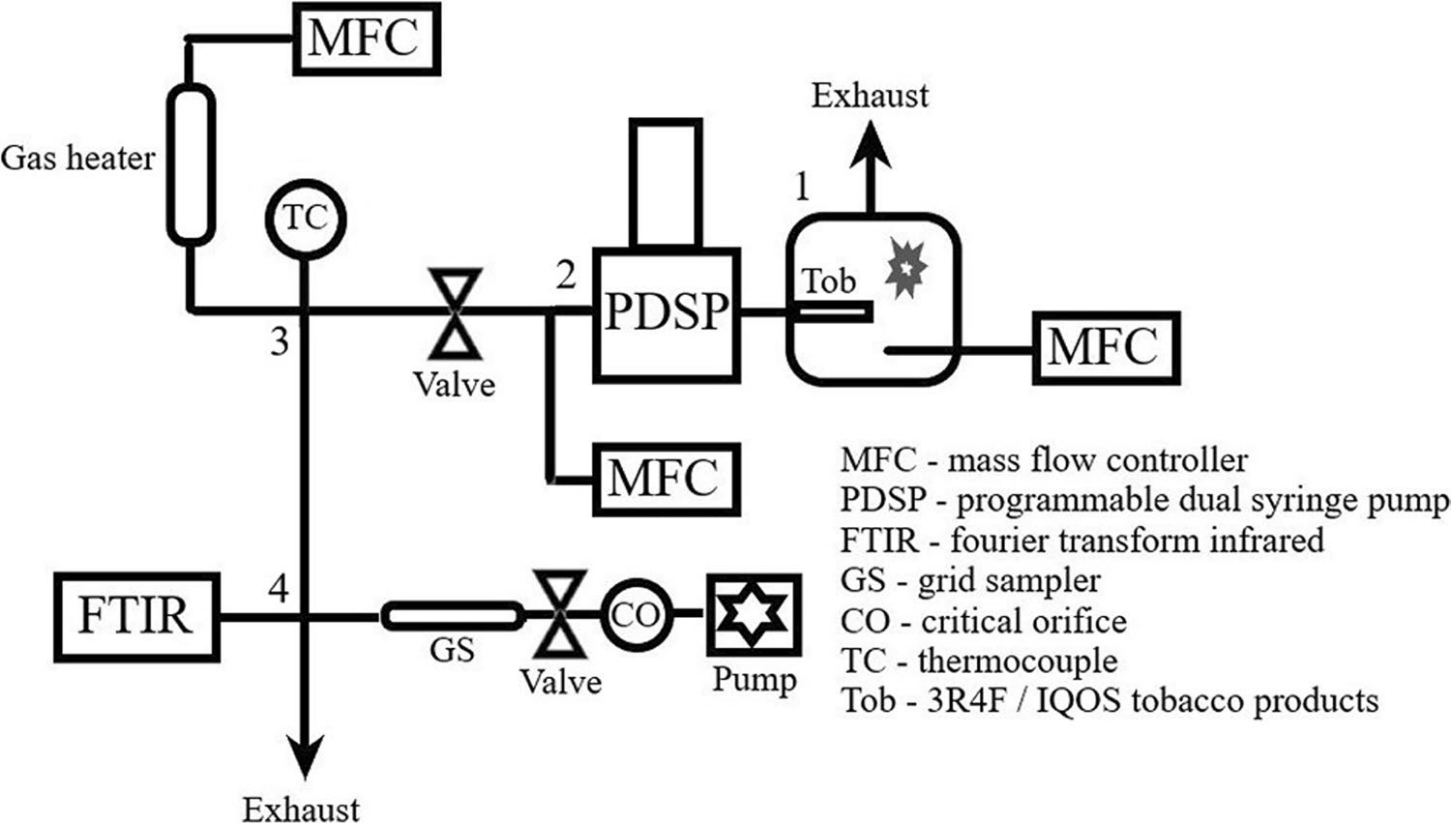
Schematics of the experimental setup. 1) PDSP inlet line connector (with a tobacco product) inside the plastic box, 2) PDSP outlet, 3) cross-connector at the location of hot dilution and 4) cross-connector splitting the sample flow for the two sampling lines and an exhaust line

The 3R4F and IQOS products were tested using the experimental setup described in Fig. 1. The setup was placed inside a fume hood. The puffing device (programmable dual syringe pump PDSP, Burghart Messtechnik GmbH) was positioned on a table. The PDSP cigarette/IQOS product holder (1) was placed inside a plastic box, which was used to prevent any sidestream emissions within the fume hood or laboratory room. A dry air flow of 1.5 L/min (measured with a Thermal Mass Flowmeter TSI 3063, TSI Incorp.) was directed through the plastic box via inlet and outlet lines. The outlet line was directed to the exhaust duct of the fume hood. The air flow enabled normal and also controlled operating conditions for the tobacco products during the experiments (burning/heating). Thus, the atmosphere inside the plastic box and around the tobacco product was dry air at 20 °C, with minimal particulate contaminants.

The outlet of the PDSP (2), releasing the smoke/aerosol samples in a controlled way, was located opposite to the PDSP inlet. The sampling line was made of the stainless-steel parts and connected to the PDSP outlet. The sampling line consisted of a cross-connector (1/4″ size), which enabled to feed a carrier air flow (1.0 L/min) to the setup and an additional exhaust line for the gas flow in case it would be needed (currently not in use). The smoke/aerosol sample (0.055 L per puff) was diluted with the carrier air flow. Subsequently, the sample was transported via a line (1/4″ size) with an on/off valve connected to the (3) second cross-connector (1/2″ size). The hot dilution at temperatures ranging from 20 to 350 °C was performed at this point, with a gas heater connected to one of the inlets of the cross-connector. Another connection was used to measure the gas phase temperature (K-type thermocouple, tip diameter 1.5 mm) of hot dilution mixture: A) hot dilution air (5–20 L/min), B) smoke/aerosol sample and C) carrier gas air (1.0 L/min), going downstream the setup through the last connection of the cross-connector (the tip of the thermocouple was inserted inside the entrance of the downstream line). All gas flows for the setup were controlled with mass flow controllers (Brooks S5851, Brooks® Instrument). Finally, the diluted sample entered (4) another cross-connector (1/2″ size). At this point, the sample flow was split to the gas phase sampling line to collect particles on a Transmission Electron Microscopy (TEM) grid with a suction sampler (0.5 L/min, flow controlled with a critical orifice and pump) and to the FTIR line for online analysis of gaseous compounds (0.85 L/min). The rest of the flow was directed through the last channel of the cross-connector to the exhaust duct of the fume hood.

The PDSP device was operated under the Health Canada Intense (HCI) regime (Health Canada 2000): 55 mL per puff, puff duration 2 s, 2 puffs per minute (once every 30 s). When testing the 3R4F cigarettes, 10 puffs were generated per cigarette. Filter ventilation holes of the cigarette were blocked with a sticker as per the HCI protocol. For IQOS, the number of puffs was 12 per heated tobacco stick.

Some of the TEM grids with the collected particle sample were placed in an aluminium foil covered alumina holder in a furnace with a stainless steel furnace tube. The TEM grids were heated at 200 °C for an hour in a dry air flow of 0.4 L/min. It enabled the vaporisation of low boiling point species and the subsequent analysis of residual deposits.

### Analysis methods

The speciation of gaseous compounds and their concentration in the gas phase was analysed using FTIR (Fourier Transform Infrared, Gasmet DX4000 portable gas analyzer) spectroscopy, which enabled the simultaneous online monitoring of multiple gaseous species in the gas flow. The main limitation of this technique is that diatomic elements such as O_2_ or noble gases cannot be detected as they do not absorb light in the infrared spectrum. The flow rate of the gaseous samples through the FTIR sampling cell was set at 0.85 L/min. To prevent the condensation of water or other corrosive gases in the sampling cell, it was heated to 180 °C. In order to reduce measurement variability, the FTIR data were averaged for an aerosol sampling time of 20 s (100 ms for individual scans).

During the FTIR measurements, a membrane filter (47 mm in diameter, MilliPore, Mitex™ PTFE, pore size 5 µm) was inserted upstream of the FTIR inlet to capture any solid particles and liquid droplets from the smoke/aerosol samples (prior to the analysis of gaseous species in the gas flow). The filters were weighed before and after the FTIR measurement to record the mass of accumulated particulate matter.

The particulate matter of the smoke/aerosol samples was collected on a 400-mesh perforated carbon film-coated copper TEM grids (Agar Scientific), see for example Fig. 9, in view of analysing them using SEM and TEM techniques. The particulate matter sampling was performed directly from the gas flow by passing a sample flow of 0.5 L/min through the grid. The main collection mechanism for particulate matter was diffusion to the grid surface (Ogura et al. 2014). The use of TEM grids allowed analysis of the same specimen using both the SEM and TEM techniques. The elemental X-ray analysis of carbon and copper is somewhat hampered due to the background counts arising from the carbon foil and copper grid.

The morphology and elemental composition of the particulate matter samples were analysed using SEM (Zeiss Crossbeam 540) and TEM (TALOS™ X200F). The X-ray analysis (EDX) in SEM and TEM was performed using a 30 cm^2^ silicon drift X-ray detectors (SSD, SEM: EDAX Elite Octane™, TEM: Bruker Super X 4-detector assembly). The SEM and TEM X-ray data (TEM operated in Scanning mode, i.e. in STEM mode) were collected in spectrum imaging (SI) mode. The SI mode produces data cubes where an X-ray spectrum is saved for each pixel in the image. The major advantage for using SI data sets is the availability of versatile methods for off-line data analysis. The pixel resolution for SI data set images was set to 256 × 256 pixels. In STEM mode the detector used for imaging was the so called “HAAD-detector” (High-angle annular dark-field) that is used for Z-contrast imaging (Z-contrast refers to atomic number contrast). Z-contrast imaging in STEM enables the efficient detection of small, nanometre sized particles on TEM grids. In SEM, the imaging was performed using the “In-lens” secondary electron (SE) detector, located above the objective lens in the SEM column. The In-lens detector is a high contrast device allowing, for example, fast imaging of beam sensitive samples and the detection of weak, very thin objects, such as those present in the IQOS heated tobacco aerosol specimens. The SEM was operated at an acceleration voltage between 8–10 kV for EDX analyses and 2–4 kV for imaging, while the TEM was operated at 200 kV. The probe current used in SEM and TEM during analyses was ~ 100 pA. Due to the significant amount of water and glycerol in the high mass-loading IQOS heated tobacco product samples (sampling time longer than 10 s), only the heat treated samples (sample heated up to 200 °C in air) were analysed in the TEM, in order to maintain operating vacuum conditions in TEM column and to protect X-ray detectors from contamination. IQOS heated tobacco samples with shorter aerosol sampling times (i.e. 3–8 s) could be analysed without heat treatment in TEM analyses. The SEM is equipped with a plasma cleaner that was used to periodically clean the specimen chamber.

### Experiments

The gaseous and suspended particulate matter fractions of smoke generated from 3R4F cigarettes, and the aerosol formed from heating the tobacco in the IQOS device were analysed. The generated smoke/aerosol was exposed to temperatures ranging from 20 to 350 °C, before sampling, to study how the organic matter evolved at different temperatures and to facilitate the analysis of possible residual solid particles.

In case of the gaseous fraction, part of the smoke/aerosol was directed through a filter to collect the particulate matter as described above. The filter was located at the inlet of a FTIR spectrometer. Subsequently, the gaseous fraction was analysed using the FTIR spectrometer. From the measurements, the analyses revealed the nature of the gaseous compounds and their concentration levels in the smoke/aerosol. The FTIR filters were also weighed to obtain the mass of the airborne particulate matter. Simultaneously, part of the suspended particulate matter was directed through a TEM grid to collect potential liquid droplets and solid particles. Afterwards, the grid was analysed using SEM, TEM, and EDX spectroscopy to determine the morphology of the particulate matter and elemental composition. After the particulate matter collection, some of the grid samples were heated in a furnace in an air atmosphere to vaporise the excess of organic material originated from the tobacco smoke/aerosol. The residual deposits were analysed using SEM and TEM.

## Results

### FTIR analysis of gaseous compounds

The gaseous compounds in 3R4F cigarette smoke and the IQOS aerosol were analysed using FTIR spectroscopy. For each data set, the background was subtracted to obtain the net amount of gases coming from the smoke/aerosol samples. The average results were calculated based on the analysis of the first 3 to 4 cigarettes/IQOS tobacco sticks used out of the 10 to 20 tested. This procedure was required due to the time-dependant mass built-up on FTIR filters/setup surfaces, which reduced the amount of smoke/aerosol transported to the FTIR analysis cell. It must be noted that all the gaseous species measured could be quantified if their concentrations were above 1 ppm.

Interestingly, the nicotine concentration seemed to decrease, and was temporally shifted, when increasing numbers of IQOS tobacco sticks were tested. In fact, for the first IQOS tobacco sticks, the nicotine peak appeared approximately at the same time as other identified compounds. Over the use of several IQOS tobacco sticks, the nicotine peak was increasingly time-shifted in comparison to the appearance of other compounds. This effect was not observed for 3R4F cigarette smoke. This delay in the appearance of nicotine was likely due to the build-up of nicotine deposits on the experimental setup surfaces during the course of IQOS testing, and its subsequent re-evaporation. Also, the nicotine peak was observed to be sharper for the IQOS aerosol compared with 3R4F cigarette smoke, and therefore upper limit values are provided.

**Fig. 2:**
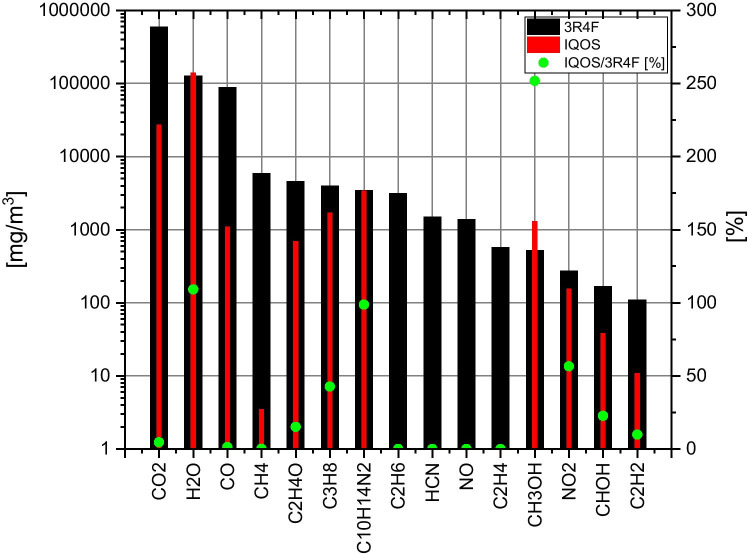
Comparison of gas mass concentrations (mg/m^3^) for 3R4F smoke and IQOS aerosol at 20 °C. The concentration ratio IQOS / 3R4F [%] is displayed

The average concentration of gaseous compounds (corrected for dilution) in 3R4F cigarette smoke and in IQOS aerosol are compared in Fig. 2 (see also Table 1). Based on the FTIR analyses (mg/m^3^ unit) of 3R4F smoke at 20 °C, the main identified gaseous components (> 1000 mg/m^3^) were carbon dioxide (CO_2_), water (H_2_O), carbon monoxide (CO), methane (CH_4_), acetaldehyde (C_2_H_4_O), propane (C_3_H_8_), nicotine (C_10_H_14_N_2_), ethane (C_2_H_6_), hydrogen cyanide (HCN) and nitrogen monoxide (NO). Other components at low concentrations were: ethylene (C_2_H_4_), methanol (CH_3_OH), nitrogen dioxide (NO_2_), formaldehyde (CHOH), and acetylene (C_2_H_2_). In the IQOS aerosol at 20 °C, the main identified gaseous components (> 1000 mg/m^3^) were water (H_2_O), carbon dioxide (CO_2_), nicotine (C_10_H_14_N_2_), propane (C_3_H_8_), methanol (CH_3_OH), and carbon monoxide (CO). Other components with low concentrations were as follows: acetaldehyde (C_2_H_4_O), nitrogen dioxide (NO_2_), formaldehyde (CHOH), acetylene (C_2_H_2_) and methane (CH_4_). Nitrogen monoxide (NO), hydrogen cyanide (HCN), ethane (C_2_H_6_), and ethylene (C_2_H_4_) were below the detection limit of FTIR.

**Table 1:** Gas mass concentration average values (mg/m^3^) and standard deviation (SD) from Fig. 2. In addition, the corresponding emission results of gases per product (cigarette or tobacco stick) and per puff are presented

	3R4F				IQOS			
	mg/m^3^	SD	mg/cig	mg per puff	mg/m^3^	SD	mg/stick	mg per puff
CO_2_	591,000	125,000	328	32.8	27,100	6460	18.1	1.5
H_2_O	128,000	17,560	70.9	7.09	139,000	8540	92.9	7.74
CO	88,300	16,600	49.1	4.91	1090	58.5	0.727	0.0606
CH_4_	5890	1420	3.27	0.327	3.44	4.38	0.0023	0.000191
C_2_H_4_O	4570	1010	2.54	0.254	691	36.5	0.461	0.0383
C_3_H_8_	3940	1150	2.19	0.219	1680	103	1.12	0.0935
C_10_H_14_N_2_	3450	120	1.92	0.192	3400	116	2.27	0.189
C_10_H_14_N_2_^a^	-	-	-	-	-	-	1.33	0.111
C_2_H_6_	3140	687	1.75	0.175	0	0	0	0
HCN	1490	349	0.826	0.0826	0	0	0	0
NO	1370	540	0.762	0.0762	0	0	0	0
C_2_H_4_	572	109	0.318	0.0318	0	0	0	0
CH_3_OH	517	258	0.287	0.0287	1300	99.1	0.868	0.0724
NO_2_	272	65.3	0.151	0.0151	154	11.1	0.103	0.00855
CHOH	167	52.1	0.0927	0.00927	37.8	10.9	0.0252	0.0021
C_2_H_2_	109	29.5	0.0605	0.00605	10.7	5.35	0.00713	0.000595

^a^IQOS transported mass concentration of nicotine on average, otherwise the IQOS nicotine mass transport results have been given based on the upper limit value. (The 3R4F transported mass concentration of nicotine is only reported based on the average value.)

In general, the concentrations of gaseous compounds were found to be lower in IQOS aerosol compared with cigarette smoke, by factors larger than 1.8. However, nicotine concentrations remained at the same level in both cases (ca. 0.192 mg and 0.189 mg of nicotine for one puff when using 3R4F and IQOS, respectively). The concentration of methanol was notably higher for IQOS. The carbon dioxide concentration was notably higher for the 3R4F product. The CO_2_ emissions in IQOS aerosol were derived from the torrefaction (mild-pyrolysis) processes (Cozzani et al. 2020). Water concentrations were practically at the same level for both products. The concentrations of CO_2_ and H_2_O for both products were high in comparison to other gaseous compounds. Although the emissions of CO_2_ and H_2_O primarily originated from the tobacco products, a fraction of the emissions was likely attributed to the room air flowing through the 3R4F and IQOS products during the testing. In fact, the room air was introduced into the system when the 3R4F or IQOS products were changed between testing. The corresponding effect of room air (at 20 °C, theoretical maximum relative humidity of 100%) on the emission results would be as a maximum approximately 1800 mg/m^3^ for CO_2_ and 1700 mg/m^3^ for H_2_O. Therefore, CO_2_ and H_2_O results were not attributed solely to tobacco emissions, although the contribution from the room air was insignificant.

**Fig. 3:**
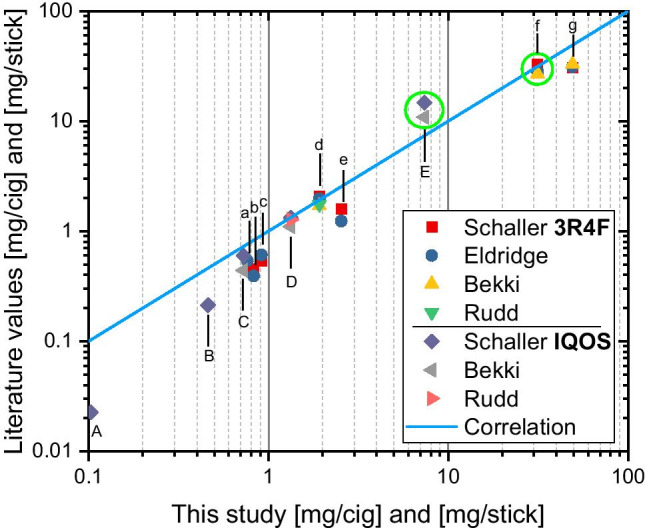
Comparison of the gaseous and particulate matter emissions from 3R4F and IQOS. The values are extracted from the results of this study (Table 1) and literature (Table 2). The comparison of particulate matter emissions is marked with green circles in the figure. **IQOS** A: NO_x_, B: C_2_H_4_O, C: CO, D: C_10_H_14_N_2_, E: TPM; **3R4F** a: NO, b: HCN, c: NO_x_, d: C_10_H_14_N_2_, e: C_2_H_4_O, f: TPM, g: CO

A summary of values found in the literature for gaseous and particulate matter emissions is given in Table 2. These values were compared with the values from the current study (extracted from Tables 1 and 3) and presented in a scatter plot in Fig. 3. Particulate matter results are further discussed in the following chapter.

**Table 2:** Summary of values found in the literature (Schaller et al. 2016; Eldridge et al. 2015; Bekki et al. 2017; Rudd et al. 2019) for gaseous and particulate matter emissions from 3R4F and IQOS. Uncertainties in the values are not shown

Ref	This study	Schaller	Eldridge	Bekki	Rudd	This study	Schaller	Bekki	Rudd
Product	3R4F					IQOS			
	mg/cig	mg/cig	mg/cig	mg/cig	mg/cig	mg/stick	mg/stick	mg/stick	mg/stick
H_2_O	70.9	13.3	15.9	10.1	-	92.9	39.4	33.1	-
CO	49.1	30.7	31.2	33.0	-	0.727	0.598	0.44	-
C_2_H_4_O	2.54	1.59	1.24	-	-	0.461	0.213	-	-
C_10_H_14_N_2_	1.92	2.09	1.97	1.7	1.76	1.33	1.32	1.10	1.27
HCN	0.826	0.451	0.392	-	-	-	0.00378	-	-
NO	0.762	-	0.540	-	-	-	-	-	-
NOx	0.913	0.541	0.608	-	-	0.103	0.0226	-	-
TPM^a^	31.4	33.0	28.2	26.8	-	7.38	14.7	10.9	-

^a^TPM (total particulate matter), water is not included in the aerosol transport results

**Table 3:** Experimental conditions for seven tests. The smoke/aerosol mass load on filters (located upstream the FTIR inlet) and gas phase mass concentrations. The mass accumulated on filters contains droplets, solid particles, tar and other possible gas phase compounds. Note: the weights were obtained without further conditioning for the studied experimental conditions—not the dry mass

Test	1	2^e^	3	4	5	6	7
Product	IQOS	IQOS	IQOS	3R4F	3R4F	3R4F	3R4F
Temperature [°C]	20 to 350	20 to 350	20	20	20	20	20
FTIR flow rate [l/min]	0.85	0.85	0.84	0.83	0.79	0.84	0.84
Carrier gas flow rate [l/min]	1.00	0.99	1.00	0.99	0.99	1.00	1.00
Hot dilution flow rate [l/min]	16.1	1.62	16.5	16.2	1.64	16.5	16.5
PDSP^a^ puff volume [ml]	55.0	55.0	55.0	55.0	55.0	55.0	55.0
Dilution ratio (DR)^b^	308.9	47.9	316.0	310.0	48.3	316.0	316.0
Number of cigarettes/IQOS tobacco sticks	10	18^e^	17	4	0.5^c^	4	8
Puffs per cigarette/IQOS tobacco stick	12	12	12	10	10	10	10
Weight of collected smoke/aerosol on filter [mg]	1.5	10^e^	3	3	2	3	6
DR corrected smoke/aerosol mass concentration [mg/m^3^]	9090	5220	11,100	56,000	48,900	56,400	56,400
Smoke/aerosol yield per product^d^ [mg/cig] or [mg/stick]	6.06	3.48^e^	7.38	31.1	27.2	31.4	31.4
Smoke/aerosol yield per puff [mg]	0.51	0.29	0.62	3.11	2.72	3.14	3.14

^a^PDSP is a short name of a programmable dual syringe pump, which was used for the testing of cigarettes and heated tobaccos. The puff frequency was 2 per minute (puff duration 2 s, one puff per 30 s)

^b^The flow of gases/smoke/aerosol through the FTIR filter was already diluted and it corresponded the outlet concentration of the experimental setup. In order to estimate the initial concentrations of gases and aerosols, a “dilution ratio” factor has been established

^c^Due to the very low dilution flow rate, the aerosol concentration was very high and therefore, the FTIR filter clogged during the test with first cigarette. Based on FTIR online data, it was estimated that only approximately half of the 3R4F cigarette was burnt when the filter clogged

^d^10 puffs was taken for 3R4F cigarettes and 12 puffs for IQOS tobacco sticks

^e^Decreased gas/aerosol transport through the filter during the test. Likely, higher aerosol transport in reality

In comparison to previous published findings (Schaller et al. 2016; Eldridge et al. 2015; Bekki et al. 2017; Rudd et al. 2019), the values obtained showed the same trend—IQOS emissions were lower than 3R4F emissions. There were some discrepancies, for example in the case of nicotine, the yield for IQOS was roughly half of the yield obtained in this study (see Table 1). However, that is likely due to difference in reporting the results. In fact, the upper limit of nicotine yield has been emphasized here and the subsequent average was 1.33 mg per product with a standard deviation of 0.36, which correspond to a level of 1.26 mg (± 0.24) per product reported in (Schaller et al. 2016). Due to the delayed and sinusoidal yield of nicotine, the upper limit was preferred for IQOS, whereas in case of 3R4F the rectangular shaped yield favoured the use of average values.

Although the analysis of water was inaccurate due to the unavoidable leakage of room air to the system, the difference in water content between the 3R4F and IQOS products was practically the same in all studies (see Table 2). The water content was approximately 25 mg (per product) higher for IQOS. (Note, in references such (Schaller et al. 2016), the water in total particulate matter (TPM) on the filter is reported only. Thus, it is not the total water content present in the aerosol, but the water in the particulate deposit used for the calculation of the nicotine free particulate matter.) In the case of carbon monoxide emissions, the results for 3R4F smoke were significantly higher than reported in the literature. However, the CO emissions for IQOS were on the same level in the current study and in the studies from literature. The analysis method was the same in both cases, thus it indicates higher CO releases from the 3R4F in this study compared with the previously reported values. One potential explanation of the higher CO values in 3R4F smoke in this study is that most of the CO in cigarette smoke is generated by incomplete combustion and therefore strongly depends on the oxygen availability around the cigarette in the experimental setup. On the contrary, the low amount of CO generated during the use of IQOS originated from the torrefaction processes, which are independent of the oxygen availability (Cozzani et al. 2020). Otherwise, the gaseous emissions were similar in all previously reported studies, although differences were more pronounced for low concentration species.

In general, the gaseous emission values in this study were found to be higher, up to a factor of 2.2, in comparison to other studies from the literature. A possible explanation could be the result of the build-up of the deposits within the experimental setup in the course of long testing, which was demonstrated in the case of nicotine emissions for IQOS. The deposits could have contributed to a lower average transported mass concentration values in the data analysis. As mentioned above, only the values obtained in the first part of the testing (3 or 4 cigarettes/heated tobacco sticks used) have been presented in this study. In addition, it is possible that the low flow rates and long flow lines of the experimental setup could have further enhanced the deposition effect. Another issue may have been the use of stainless steel for the flow lines in this investigation. The contribution of uncertainties associated with the different analytical methods, for example, the accuracy of FTIR references for the specific species and concentrations to be analysed, are also potential sources for variation between the results reported here and other authors. In particular, the species with low concentration in the gas phase may have resulted in high experimental variability.

Hot dilution in the range 20 to 350 °C did not show a significant effect on the gaseous compound concentrations. The hot dilution temperature remained lower than the actual operation temperatures of 3R4F cigarettes and IQOS heated tobacco products. Therefore, no significant reactions were observed, especially in the rather short transfer time of ca. 1 to 2 s within the hot zone of the experimental setup, being close to the set-point temperature of hot dilution. The likely effect of heating was to enhance the transport of compounds in the downstream part of experimental setup, since the condensation and deposition phenomena were suppressed. Possibly, the dilution flow was also enhancing the transport of compounds by reducing their concentration, reactions with the surfaces and residence time within the experimental setup.

### Transport of particulate matter during FTIR analysis

**Fig. 4:**
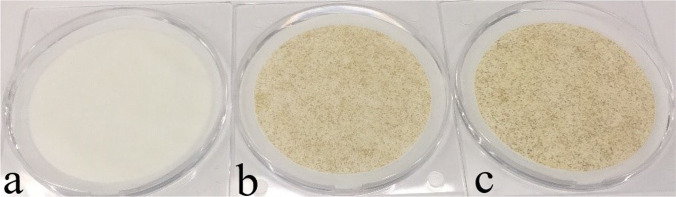
Picture showing the filters with the collected particulate matter of IQOS aerosol and 3R4F smoke. Filter (**a**) was obtained after using 17 IQOS heated tobacco sticks. Filters (**b**) and (**c**) were obtained after smoking 4 and 8 3R4F cigarettes, respectively. The dilution ratio was the same for all tested samples

During the FTIR measurements for 3R4F smoke and IQOS aerosol, a membrane filter was inserted upstream of the FTIR to separate the solid particles and droplets of smoke/aerosol from the aerosol/smoke sample. A visible difference was observed in the colour of the collected particulate matter from the IQOS aerosol (Fig. 4(a)) and from 3R4F cigarette smoke (Fig. 4(b) and (c)), which was also reported in the study by (Schaller et al. 2016). The deposition of 3R4F smoke was characterized by dark-yellow and brown colours, whereas the deposited IQOS aerosol was a pale-yellow tone. The filters were also analysed by weighing the accumulated mass. The experimental conditions and the particulate matter accumulated on filters are summarized for seven tests in Table 3.

Based on the results given in Table 3, the IQOS aerosol mass concentration ranged from 5 to 11 g/m^3^. In the case of 3R4F cigarette, the smoke mass concentration ranged from 49 to 56 g/m^3^. Therefore, IQOS emissions resulted in approximately 5–10 times lower captured mass in comparison to 3R4F cigarette.

The results were compared with the previous work of (Schaller et al. 2016; Eldridge et al. 2015; Bekki et al. 2017), see Table 2. In order to facilitate the comparison, the water content was subtracted from the aerosol transport results (TPM = total particulate matter) of these authors. The current values were showing the same trend, IQOS emissions were lower compared to 3R4F emissions (see also Fig. 3). In fact, the results for 3R4F were nearly at same level in all reported studies, whereas for IQOS the aerosol mass yield was somewhat lower (although within the rather large uncertainties reported by the authors). The water (vapour) content of IQOS aerosol was 2.3 times larger in comparison to 3R4F smoke (Schaller et al. 2016). The differences in the water content of both products and the related uncertainties seem to explain the variation between the aerosol transport results between different authors herein. In addition, based on the results of Schaller et al. (2016), a fraction of the gas phase nicotine is known to condense onto existing aerosol particulate matter, which may have resulted in increased nicotine transport via the suspended particulate matter. Therefore, the higher gas phase transport of nicotine for IQOS (see Table 1) may have resulted in a lower mass transport of aerosol particulate matter for IQOS in this study. Another possible explanation could be that the build-up of deposited aerosol within the experimental setup may have resulted in lower suspended particulate matter reaching the filter due to the high volatile of the IQOS aerosol in comparison to 3R4F cigarette smoke. To obtain sufficient aerosol mass to perform the analyses at least ten IQOS tobacco sticks were accumulated before removing the filter. Therefore, this relatively long sampling time may have led to the formation of aerosol deposits in the experimental setup leading to less particulate matter deposition on the filters. Also, the relatively low aerosol mass filter loading was found to be close to the working range of the scale. This could also have contributed to the variability in the results.

### Microscopy analysis of tobacco product materials and smoke/aerosol particulate matter

**Fig. 5:**
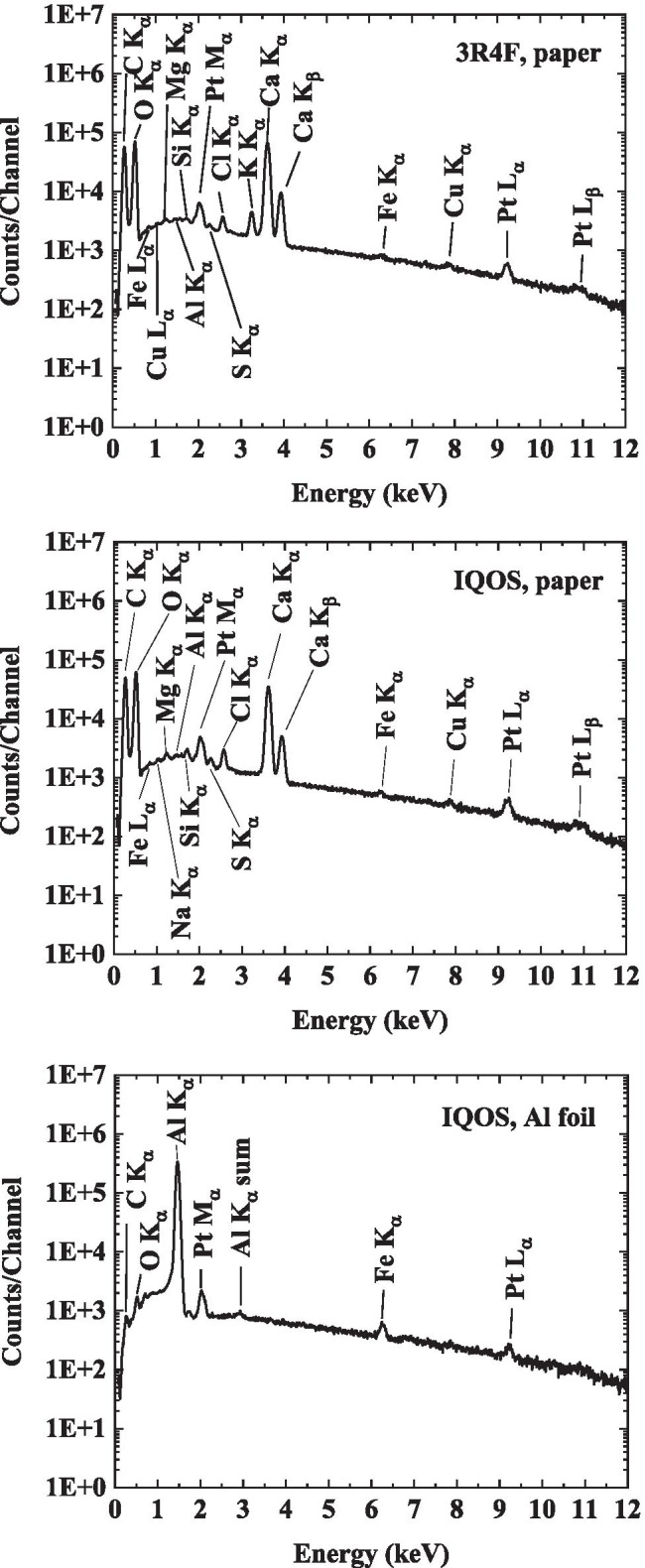
Elements observed in the tobacco wrap papers of the studied products (3R4F and IQOS). The tobacco wrap paper in IQOS tobacco sticks (middle figure) had two layers: The outer paper was similar to the 3R4F paper (top), whereas the inner layer appeared to be an Al foil (bottom). The major difference in the paper was that potassium was not observed in the IQOS paper. The calcium was found to be attributed to the paper fillers and pigments. The platinum signal is attributed to the conductive coating of the samples

**Fig. 6:**
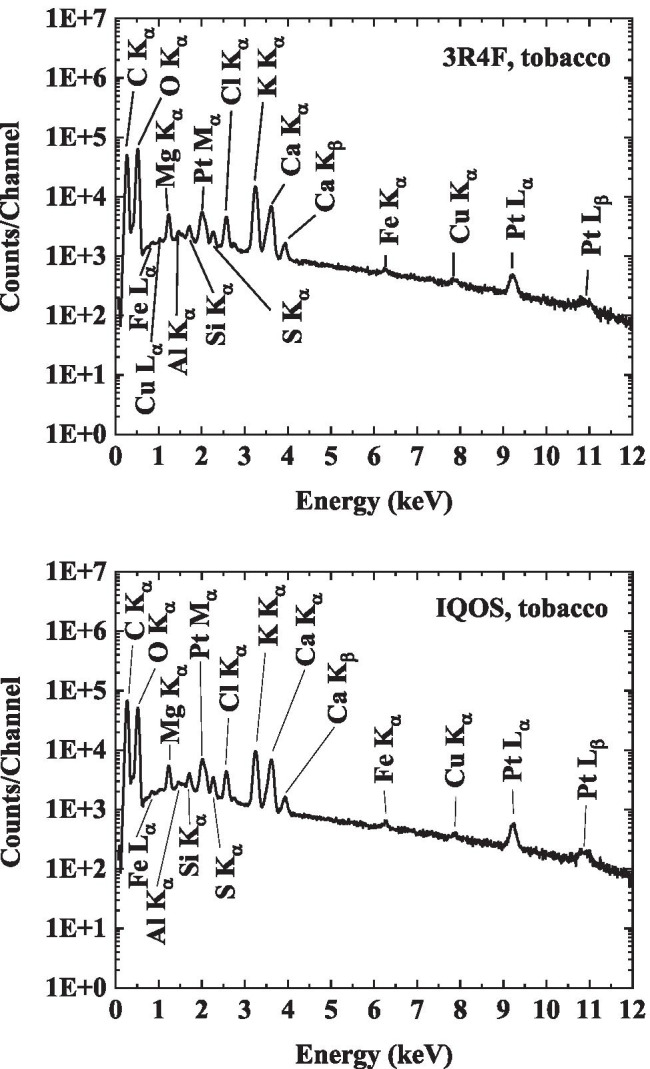
Elements observed in the tobacco material from a 3R4F cigarette (top) and from an IQOS tobacco stick (bottom). The observed elements are typical for living plant organisms. The platinum signal is attributed to the conductive coating of the samples

Materials from the 3R4F cigarette and IQOS tobacco stick were analysed using SEM and EDX to identify the possible sources of observed elemental compositions in the particulate matter samples. Both the paper encapsulating the processed tobacco material and the tobacco material itself were analysed. The samples (ca. 1 cm × 1 cm) were attached to aluminium stubs (diameter 12.5 mm) using double sided carbon adhesive tape. Prior to analyses, both the paper and the tobacco samples were coated with a platinum layer to improve charge conductivity. The measured spectra are shown in Figs. 5 and 6 for paper and tobacco, respectively.

According to SEM/EDX analysis, the IQOS tobacco wrap paper composition was quite similar to the 3R4F paper composition. A major difference in the elemental composition was found in the potassium content of paper, no potassium was observed in the IQOS tobacco wrap paper. Another remarkable difference between the tobacco paper wrap in IQOS and the paper in 3R4F cigarettes was observed. The IQOS had an aluminium foil under the paper (see bottom spectrum in Fig. 5) to prevent users from lighting the tobacco stick with a lighter.

The elements observed in the tobacco material samples of 3R4F and IQOS (see Fig. 6) included potassium, calcium and magnesium, compounds typically found in living plants (Naeem et al. 2017). These compounds, or traces of them, were also found in the particulate matter analyses as described later. The elements found are those, which are detectable by EDX analysis in SEM. Other, more sensitive methods, would potentially detect other elements, especially those at ppm levels or below (trace elements in plants). A significant proportion of the carbon detected in the IQOS tobacco substrate originated from glycerol, which is used as an additive to enhance aerosol formation (Schaller et al. 2016; Cozzani et al. 2020).

### Analysis of 3R4F smoke particulate matter

**Fig. 7:**
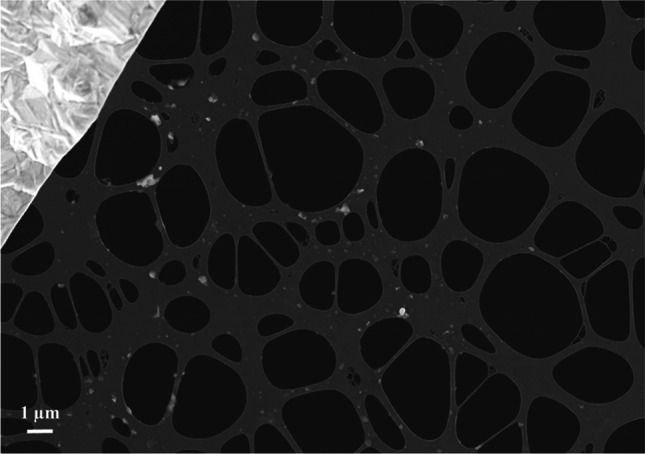
SEM micrograph of 3R4F smoke sample. Several individual particles are visible as pale grey coloured areas on the carbon film. Scale bar is 1 µm, testing at 20 °C

**Fig. 8:**
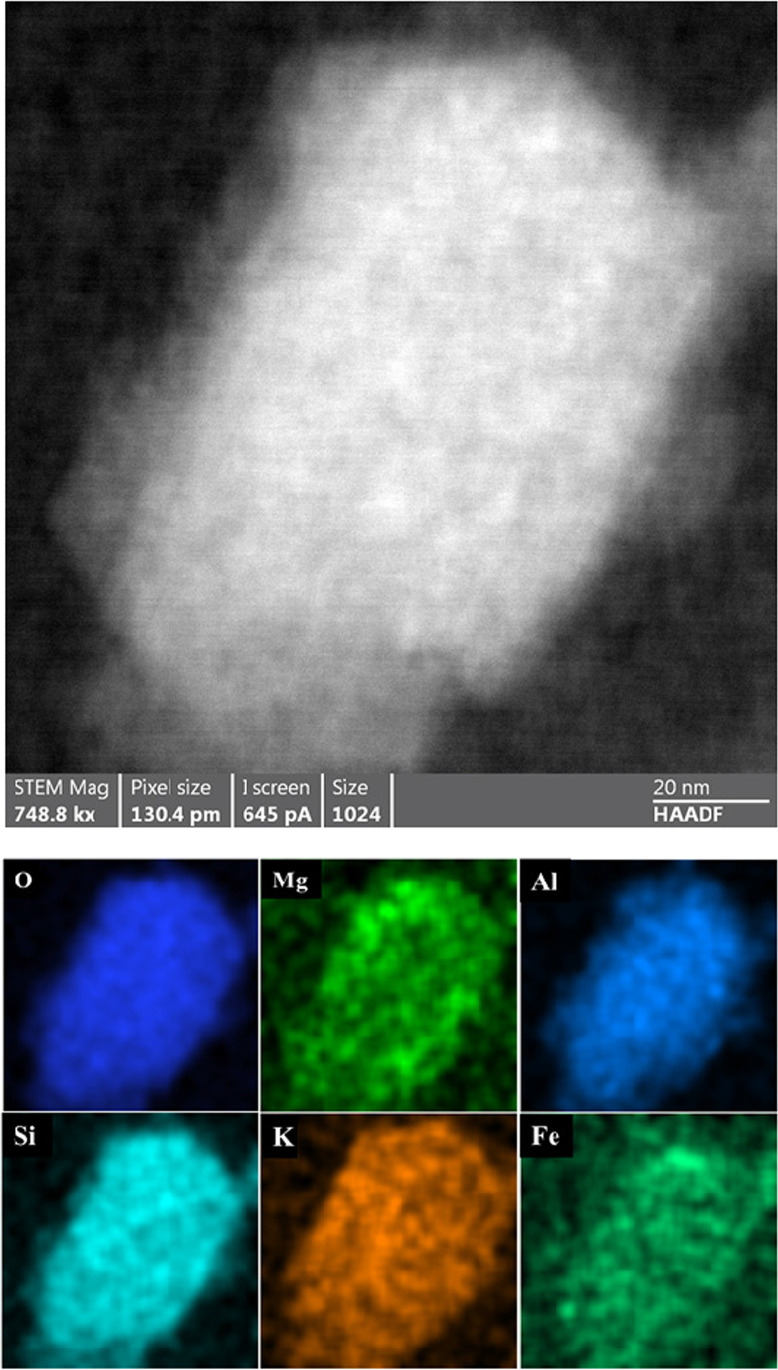
STEM analysis of an individual 3R4F smoke particle in Fig. 7. On top is a HAADF micrograph (scale bar is 20 nm, testing at 20 °C) and at the bottom are the EDX elemental maps of the observed elements (Oxygen (O), Magnesium (Mg), Aluminium (Al), Silicon (Si), Potassium (K), and Iron (Fe)). Copper is omitted as it arises from the grid. The EDX maps show typical uniform distribution of multi-elemental composition of particles observed in the in-organic 3R4F samples

**Fig. 9:**
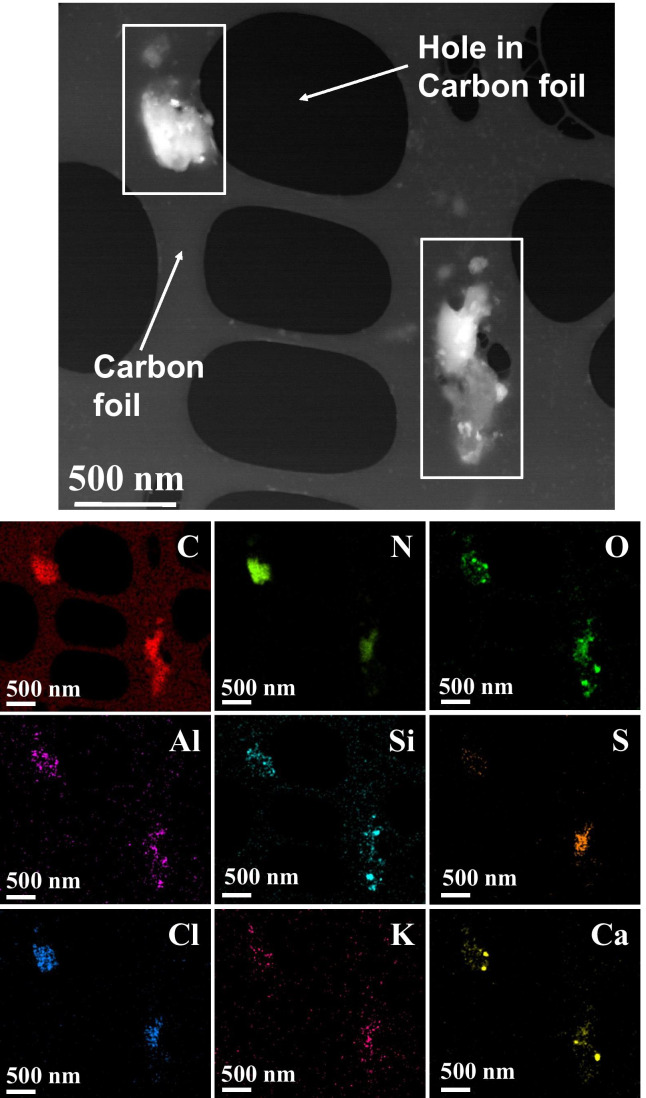
Dark field STEM micrograph and EDX elemental maps for the observed elements of a 3R4F smoke particle (scale bar is 500 nm, testing at 200 °C). Two larger carbon-rich particles are indicated with rectangles. The elemental maps show imbedded in these two larger particles several smaller particles having multi-component elemental composition. Otherwise, the elements are those found in the tobacco samples from a 3R4F cigarette (see Fig. 6)

The particulate matter from 3R4F cigarette smoke was collected on TEM grids, which were subsequently analysed using SEM/TEM/EDX techniques. A collection of micrographs and the associated analyses for 3R4F cigarette smoke samples are shown in Figs. 7, 8, and 9. Note that all samples were collected on copper grids with carbon foil, hence the copper signal observed is attributed to the grid while the perforated carbon film on the TEM grids always contributed to the observed carbon peak intensity.

Based on the STEM/EDX analyses, it was found that the 3R4F smoke particles were mainly composed of carbon (C), oxygen (O), potassium (K), and calcium (Ca). Other minor components were also detected, such as silicon (Si), chlorine (Cl), sulphur (S), nitrogen (N), and aluminium (Al). Though not detected in the particles visible in Fig. 9, traces of zinc and magnesium as well as iron were also observed in some particles in other frames. Part of the carbon and copper detected originated from the TEM grid (carbon film and copper grid). Iron was most likely released from the experimental setup because at higher temperatures (i.e. 150 °C to 350 °C) the amount of iron-rich particles increased.

**Fig. 10:**
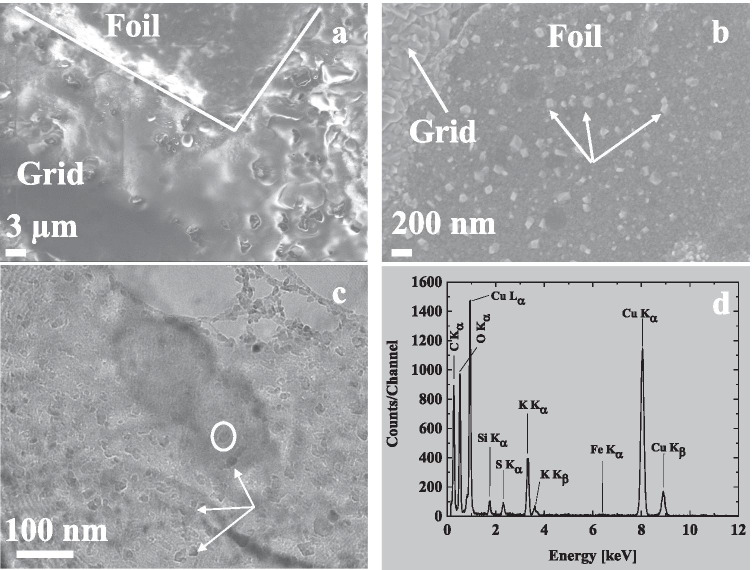
SEM micrographs (**a,b**) and TEM/EDX (**c,d**) analysis of the collected particulate matter from 3R4F smoke (sampling at high gas phase concentration of smoke), which was heated to 200 °C for an hour in a furnace in an air atmosphere to vaporize the excess carbonaceous material. The grid sample prior heating is shown in (**a**). The copper grid and carbon foil areas are indicated with lines in the figure. The whole sample is covered with featureless organic material, no individual particles are visible. In (**b**) is the SEM micrograph of the sample after heating showing several particles (some indicated by arrows). According to the TEM analyses (**c, d**), the particles (some indicated by arrows in (**c**)) composed mainly of potassium (K). The X-ray spectrum in (**d**) is measured from the particle visible inside the circle in (**c**)

In order to obtain a grid sample with a high amount of particles, the dilution ratio was decreased and a higher TEM grid loading was achieved. An SEM micrograph of this grid sample is shown in Fig. 10(a). Based on the SEM analysis, it can be concluded that the grid was covered by a thick layer of carbonaceous compounds and that no individual particles could be observed. Subsequently, the same sample was placed in a furnace at 200 °C, for an hour, in a dry air flow to remove volatile compounds. The temperature of 200 °C was chosen to avoid damaging the TEM grid. The resulting solid particle residues can be observed in SEM analyses (Fig. 10(b)). This residue was subsequently analysed using TEM/EDX (Fig. 10(c) and (d)), which showed that the particulate matter was mainly composed of crystallized potassium-rich particles embedded in carbonaceous, non-crystalline organic material.

### Analysis of IQOS aerosol particulate matter

**Fig. 11:**
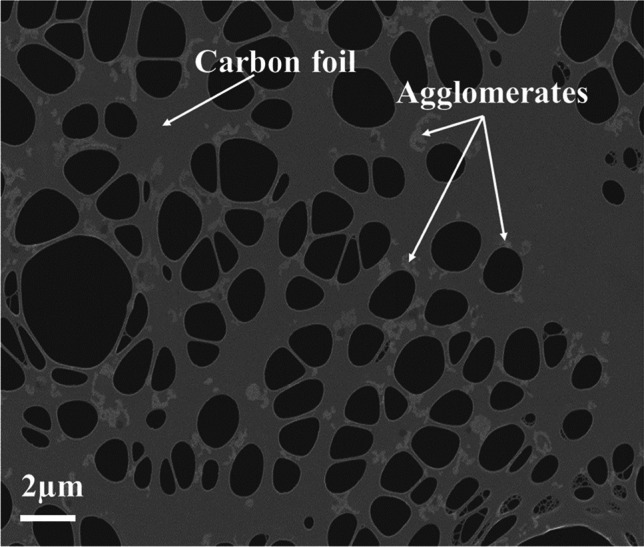
SEM micrograph of the IQOS aerosol sample collected at 20 °C. The aerosol appears as agglomerate chains (some indicated by arrows) on the carbon film

**Fig. 12:**
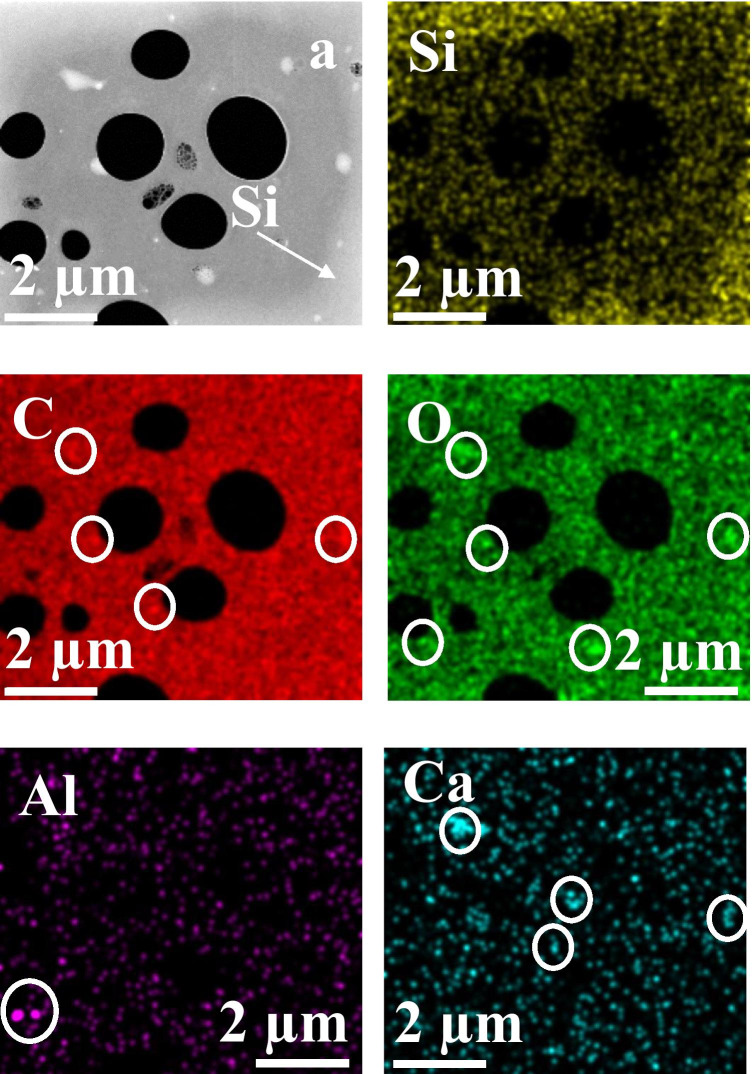
STEM/EDX elemental maps of an IQOS low mass aerosol sample. In (**a**) is a HAADF micrograph showing analysed frame after EDX mapping. The arrow in (**a**) and the corresponding silicon map indicate strong silicon diffusion along the carbon foil during the EDX mapping. The elemental maps are rather noisy. The observed particles in elemental maps are indicated by circles. Aluminium and calcium particles seem to be oxides. Silicon map is also in oxide form as there is a weak increase in oxygen content towards the edges of the frame (similar as with silicon)

The particulate matter from IQOS aerosol was collected on TEM grids for subsequent imaging and elemental composition analyses using SEM and TEM. Micrographs and elemental maps are presented in Figs. 11 to 12 for the samples collected during the tests performed at 20 °C. In Fig. 11, a SEM micrograph of the collected IQOS aerosol is shown. The deposited particulate matter had the appearance of droplet-like agglomerates, without any clear indication of primary particles. The particulate matter was somewhat beam sensitive and easily evaporated during the imaging due to electron beam interaction (heating, irradiation damage). It was not possible to obtain a clear view of elemental composition of the particulate matter using SEM because of the X-ray background arising from the TEM grid (and also aluminium background from the sample holder). The X-ray background arising from the carbon foil (carbon and oxygen as well as strong copper peak) greatly hampered the carbon and oxygen analysis. However, it is known that particulate matter is formed from vaporized organic material (i.e. tobacco) and the glycerol, which is added to the tobacco substrate in IQOS tobacco sticks to enhance aerosol formation (Schaller et al. 2016; Cozzani et al. 2020). The particulate matter shown in Fig. 11 is most likely agglomerates of the aerosol former glycerol, together with condensates of other emitted compounds, some which were observed in FTIR analyses. Upon deposition on the carbon film, the agglomerates had diffused along the carbon film, producing irregular shaped particulate material on the film.

STEM analysis of the non-heated, low mass-loading IQOS aerosol sample (test performed at 20 °C) is shown in Fig. 12. The figure a has a bright contrast close to frame edges that is due to silicon found in this sample (see the silicon map in Fig. 12). The observed amount of silicon is not typical and is most likely arising from an external source (e.g. laboratory air, experimental setup) rather than from the IQOS tobacco product. During STEM analysis, due to electron beam heating silicon is diffusing along the carbon foil towards the frame area being analysed. To reduce the amount of silicon build-up, it was necessary to use rather short collection time (ca. 20 min) for EDX mapping. The elemental maps were rather noisy making it quite difficult to identify the observed particulate matter and their composition. The observed particulate matter is indicated by circles in the maps. Similar agglomerates that are visible in the SEM micrograph of Fig. 11 partly disappeared during the STEM elemental mapping most likely due to evaporation. Some traces of Al-rich as well as Ca-rich particles were observed and the oxygen signal seemed to correspond with calcium. Otherwise, carbon and oxygen maps do not show clear features that could be attributed to the observed agglomerate structures; the contribution to the signal arising from the particulate matter on the carbon film is too weak to be detected in the carbon and oxygen maps. The carbon-rich particulate matter is most likely arising from the aerosol former glycerol additive in IQOS.

**Fig. 13:**
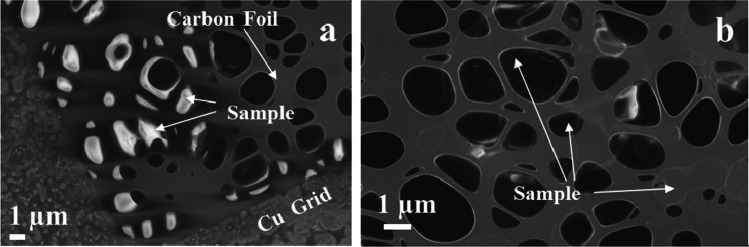
SEM micrographs of a sample of the collected particulate matter from the IQOS aerosol, which was used in the heating experiment at 200 °C. (**a**): the collected material is shown prior to heating and (**b**): after heating showing film-like structures that were hardly visible in SEM

**Fig. 14:**
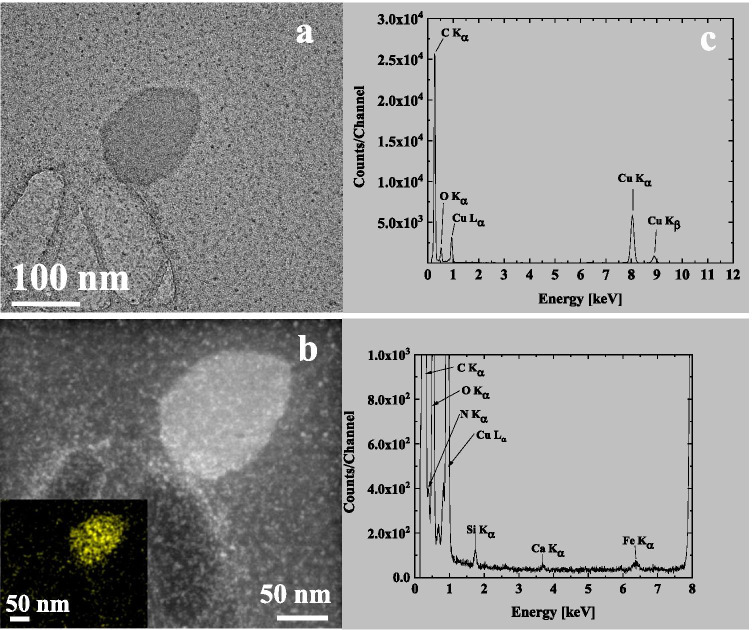
TEM micrograph (**a**) and STEM HAADF image (**b**) of a sample of the collected particulate matter from the IQOS aerosol heated at 200 °C in a dry air flow for one hour. The X-ray spectrum in (**c**), and its magnification below, show the elemental content of the sample. The deposited particulate matter in (**b**) appeared to be composed mainly of carbon and oxygen (originating from the glycerol additive) together with nitrogen (nitrogen map given in inset in b). In addition, small amounts of Si, Ca and Fe were observed in the heated sample. The (amorphous) grainy structure of the sample may be due to small Fe clusters produced during the heating

The heating experiment similar to the one for the 3R4F smoke was also conducted for the IQOS aerosol sample. However, in the sample of the collected particulate matter from the IQOS aerosol, the amount of deposited matter was much lower than in the sample of the collected particulate matter from 3R4F smoke. After the aerosol sampling, the same sample was placed in a furnace at 200 °C, for an hour, in a dry air flow to remove volatile compounds. The temperature of 200 °C was chosen to avoid damaging the TEM grid. The microscopy analyses of the IQOS aerosol sample prior to and after heating using SEM and TEM techniques are shown in Figs. 13 and 14, respectively. The IQOS aerosol sample was mainly vaporised during the heating. This shows the volatile nature of the liquid-like deposited particulate matter from the IQOS aerosol. The remaining thin layers on the carbon foil were hardly visible in the SEM images (Fig. 13(b)). Only some remaining deposits were observed, and they were of carbon and oxygen with some nitrogen (Fig. 14), originating possibly from nicotine (boiling point of nicotine is 247 °C). In addition, small amounts of Si, Ca and Fe were observed. The source of iron may have been the stainless steel materials used to convey the aerosol to the TEM grid. No potassium containing crystals were observed in the IQOS aerosol sample after the heating test, whereas the 3R4F smoke sample contained a high amount of solid potassium containing crystals. The observed inorganic particles may originate from the tobacco and the wrap paper as was shown in analysis of the paper and tobacco materials, possibly due to the air flow through the IQOS stick during testing, whereas the carbonaceous material is most likely originating from the glycerol additive part of the IQOS tobacco material (boiling point of glycerol is 290 °C).

### Discussion on the microscopy analyses of particulate matter

As observed in the microscopy analyses of the deposited particulate matter from the IQOS aerosol before heating, the majority of the deposits consisted of organic carbonaceous particulate matter, producing thick featureless layers without observable individual solid particles on the sample grids. This indicates that the deposited particulate matter was in liquid phase as suggested also by the diluted sample experiments. In contrast to the analysed collected 3R4F smoke particles from the combustion process, solid particles from the heating process were not observed in the collected IQOS aerosol particulate matter. Only some traces of calcium and aluminium were observed, most likely representing fragments of the tobacco paper and tobacco material stripped directly from the tobacco stick by the air flow during puffing as suggested by the elemental analyses in Figs. 5 and 6. Another explanation could be that the inorganic elements essential for living plant (e.g. tobacco) evaporated together with organic, carbon based chemical compounds when heated to ca. 350 °C. New particle formation could also be possible when the vapours cool down to room temperature—assuming that the supersaturation conditions for the vapours have been reached for the condensation to take place. However, this is unlikely since the concentrations of these compounds were very low. Other elemental constitutes of the tobacco material and paper were not detected in the analysed particulate matter of the IQOS aerosol, indicating that the heating of tobacco at temperature lower than required by the combustion process releases less aerosol compounds than observed for the combusted 3R4F cigarette. Traces of silicon was found to be spread all around the 3R4F and IQOS samples.

In comparison to a previous work, where the smoke passed through a thermodenuder set at 300 °C (Pratte et al. 2017), 3R4F smoke was reported to consist mainly of carbon-based material with oxygen. In addition, potassium and chlorine were found. Also, to a lesser extent, traces of aluminium, sulphur, and silicon were detected. These findings support the outcomes of this study. For the IQOS aerosol, an absence of solid organic particles in the IQOS mainstream aerosol was indicated by (Pratte et al. 2017). In the current study, carbon-based particulate matter was observed in the aerosol sample. The deposited IQOS aerosol particulate matter appeared to be featureless without individual particles visible. Moreover, the particulate matter was beam sensitive, evaporating under the electron beam, which suggests that the particulate matter in the IQOS aerosol is in liquid phase and verifies the absence of solid organic particles.

## Conclusions

The objective of this work was to compare the 3R4F cigarette smoke and the IQOS aerosol for gas phase constituents and suspended particulate matter. Out of the fifteen gaseous compounds analysed, all were identified in the smoke of 3R4F from the combustion, whereas the lower temperature heating in IQOS resulted generally in a notable reduction of gaseous emissions and species, within the limits of detection of the analysis method. As an interesting detail, it seemed that the maximum concentration of the released nicotine within a puff was practically the same for both types of products, although the average concentration was lower for the IQOS aerosol. The results indicated significantly lower suspended particulate matter in the IQOS aerosol in comparison to the 3R4F cigarette smoke.

In the analysis of particulate matter, several elements were observed in the 3R4F smoke. The combustion of tobacco released volatile carbon-containing materials that partially formed liquid-like particulate matter due to gas phase/gas phase condensation (homogeneous nucleation) and gas phase/solid particles condensation (heterogeneous nucleation). In this combustion process, soot (carbon) particles are formed as a result of incomplete combustion. However, they could not be differentiated directly from the overall carbonaceous materials in the smoke. Therefore, the gas phase and particulate matter analyses were conducted. Also, solid materials originating from the non-combustible minerals were observed such as potassium, calcium, and magnesium from tobacco combustion. In contrast, the heated tobacco in the IQOS product generated an aerosol with substantially different physico-chemical properties in comparison to cigarette smoke. The analysed particulate matter was found to have a featureless form and containing mainly carbon and oxygen. Traces of silicon was also observed. The particulate matter in the aerosol was found to be liquid-like and volatile under the electron beam. It is likely, that the carbon in the particulate matter originated mainly from the aerosol former glycerol, that evaporated from the tobacco material during heating. In general, silicon was found to be spread all around the 3R4F and IQOS samples. The trace amounts of silicon likely originated from the tobacco material, which was verified to contain silicon for both 3R4F and IQOS, being a typical inorganic element in the form of silica or aluminium silicates in tobacco leaves (see Pappas et al. 2016).

The current results were generally in good agreement with the published data. The data demonstrated that IQOS generates substantially lower emissions in comparison to cigarette smoke. This indicates that IQOS users are significantly less exposed to gaseous and particulate matter components compared to cigarette users. However, some discrepancies between the numerical results were found and the impact of deposits formation within the experimental setup used was considered. In this study, the long duration of the tests with several cigarettes or heated tobacco sticks used in a row led to a formation of deposits in the experimental setup, which could have an effect on the results. In addition, there was a delay in the appearance of the nicotine peak in comparison to other species for the IQOS aerosol. This behaviour could also lead to an erroneous interpretation of the nicotine mass concentration generally.

The comparison of these tobacco products will be continued and the next study will focus on online gaseous and aerosol emissions analyses to deepening our understanding on the physicochemical properties of 3R4F smoke, IQOS and e-vapor products aerosols.

## Data Availability

Most of the data generated or analysed during this study are included in this published article. In addition, the datasets used and/or analysed during the current study are available from the corresponding author on reasonable request.
